# The Conception of Test Fields for Fast Geometric Calibration of the FLIR VUE PRO Thermal Camera for Low-Cost UAV Applications

**DOI:** 10.3390/s22072468

**Published:** 2022-03-23

**Authors:** Anna Fryskowska-Skibniewska, Paulina Delis, Michal Kedzierski, Dominik Matusiak

**Affiliations:** Department of Imagery Intelligence, Faculty of Civil Engineering and Geodesy, Military University of Technology, 00-908 Warsaw, Poland; anna.fryskowska@wat.edu.pl (A.F.-S.); michal.kedzierski@wat.edu.pl (M.K.); dominik.matusiak@gmail.com (D.M.)

**Keywords:** calibration target, camera calibration, UAV, thermal camera, image processing, interior orientation parameters

## Abstract

The dynamic evolution of photogrammetry led to the development of numerous methods of geometric calibration of cameras, which are mostly based on building flat targets (fields) with features that can be distinguished in the images. Geometric calibration of thermal cameras for UAVs is an active research field that attracts numerous researchers. As a result of their low price and general availability, non-metric cameras are being increasingly used for measurement purposes. Apart from resolution, non-metric sensors do not have any other known parameters. The commonly applied process is self-calibration, which enables the determining of the approximate elements of the camera’s interior orientation. The purpose of this work was to analyze the possibilities of geometric calibration of thermal UAV cameras using proposed test field patterns and materials. The experiment was conducted on a FLIR VUE PRO thermal camera dedicated to UAV platforms. The authors propose the selection of various image processing methods (histogram equalization, thresholding, brightness correction) in order to improve the quality of the thermograms. The consecutive processing methods resulted in over 80% effectiveness on average by 94%, 81%, and 80 %, respectively. This effectiveness, for no processing and processing with the use of the filtering method, was: 42% and 38%, respectively. Only high-pass filtering did not improve the obtained results. The final results of the proposed method and structure of test fields were verified on chosen geometric calibration algorithms. The results of fast and low-cost calibration are satisfactory, especially in terms of the automation of this process. For geometric correction, the standard deviations for the results of specific methods of thermogram sharpness enhancement are two to three times better than results without any correction.

## 1. Introduction

Research on thermal cameras allowed us to realize that the possibilities to apply infrared imaging are unlimited, in any field of science. As a result of the successful applications of thermal infrared, the parameters of the constructed devices have improved continuously. Contemporary thermal imaging cameras have become increasingly popular recently due to their functionalities and easy operation. The dynamic development in technology, especially optoelectronics, has led to the growing importance of thermal visual imaging also in the fields of photogrammetry and remote sensing [1]. It is useful, in particular, in determining the distribution of temperatures on an object, e.g., during thermographic building inspections, as well as for the inspections of inaccessible heating facilities or power distribution networks [2]. On the other hand, in photogrammetry, Unmanned Aerial Vehicles (UAV) have been extensively used for mapping and 3D modeling in the geomatics field [3]. These platforms, equipped with RGB cameras, now represent a common alternative to traditional aerial manned photogrammetry. Another field that is enjoying growing interest is the combination of thermal cameras and unmanned platforms that offer such products as 3D models or orthoimages for the purposes of forestry and agriculture [4,5], archaeology [6,7] architecture [8,9,10], cultural heritage [11,12], environmental surveying [13,14,15,16,17,18,19,20,21,22,23,24,25], emergency management and traffic monitoring [26,27].

As far as low altitude photogrammetric studies are concerned, one of the necessary stages of data processing is geometric camera calibration. The geometric calibration of a camera lens relies on determining the value of interior orientation elements, i.e., focal length, coordinates of principal point in image coordinate system, radial and tangential distortion, skew [28,29]. The dynamic development of photogrammetry has resulted in the emergence of numerous calibration methods. Unfortunately, none of them enables direct measurement of the elements of interior orientation. Instead, other physical properties that are closely linked to the determined parameters are measured. On the other hand, the determining elements of interior orientation are the sources of errors that affect the geometric accuracy of the final photogrammetric studies [30,31].

As far as thermography is concerned, calibration is rather associated with the need to determine the radiometry of sensors. Geometric applications are still rare; hence, cameras closely linked to the determined parameters lopers and suppliers still show little interest in photogrammetric techniques. However, with the increasing resolution of thermographic cameras, the geometric processing of the image data will become more important [28]. 

In contrast to methods used for sensors in the visible spectrum, calibration field tests for thermal sensors have to take into account such characteristics as emissivity and thermal conductivity and require calibration targets with distinctive features in the IR part of the spectrum.

Numerous researchers have already attempted to determine a mathematical model of a camera that would allow for the application of thermal sensors in photogrammetry [28,29,30,31,32,33]. Researchers calibrate infrared sensors with the use of various test field patterns [29,34,35,36]. There are also other studies of geometric calibration of thermal–infrared cameras (but not in low-cost applications) [37,38,39]. 

Saponaro et al. in [40], proposed calibration using test fields for visible spectrum cameras. Nevertheless, this approach requires heating and an image processing step to enhance the corners of the images [34]. Cheng et al. [41], proposed a solution based on a black-and-white checkerboard that was heated with an infrared heater. Due to the lack of possibility to heat the whole board evenly, it was impossible to obtain thermograms of sufficiently good quality for calibration purposes [42]. Additionally, Ref. [42] presents another unique method that employs micro-electro-mechanical sensors (MEMS). However, as the size of the sensors was too small, it was impossible to conduct a sufficiently accurate measurement of differences in temperature between the board and the point heat sources. Luhmann [28], presented his own concept of an active calibration field. It consisted of a black plastic board with installed lightbulbs that emitted heat and light. The problems with the precise measurement of the center of the radiation source were caused by a decrease in the accuracy of the determining elements of interior orientation. Probably the most interesting concept was proposed by Ursine et al. [43], who constructed a device consisting of a low-emissivity base (a copper board) and high-emissivity targets that were spray-painted on the board, ensuring high accuracy of the test. Similar solutions using wooden plates, lamps, and aluminum foil or high emissivity spray ink, are presented in [44,45,46].

The quality of the geometric calibration of UAV cameras (visible and thermal range) is more or less reflected in the quality of the products developed from their images. Examples of research work in this field can be found in [47,48,49].

In an attempt to solve the geometric calibration issues of thermal cameras that had been highlighted in the studies referenced above, the authors propose a novel concept for calibration tests and image processing methodology. The authors of the present study propose an appropriate selection of the shape and model of the calibration test pattern. We suggest also an analysis of the influence of the field itself and external factors on the results of geometric calibration of a thermal camera dedicated for UAV platforms. The concept involves the construction of a low-cost, passive flat test field that will consist of a high-emissivity background and low-emissivity targets that use the phenomenon of thermal conductivity of certain materials.

The purpose of this work was to analyze the possibilities of geometric calibration of a thermal UAV camera using the proposed test field patterns and materials in various testing conditions. Consequently, the research aim was to determine the impact of the calibration test structure/pattern and external data acquisition conditions on the quality of the calibration test’s image.

Finally, geometric calibration of the thermal UAV camera (for one chosen calibration test) was evaluated to answer the question about the technical conditions of the geometric calibration process. The specific research tasks had to be defined to perform measurements properly in order to achieve the formulated research goals and to evaluate functional relationships between the camera interior orientation elements and the test field patterns in various testing conditions.

The authors of the paper have formulated the following specific objectives as a part of the research stages:

1. Preparation of suitable calibration patterns and materials;
2. Determination of the operating conditions and their influence on the image quality (in the context of unambiguous identification of calibration test features);
3. Validity and accuracy verification of the methods used for test image enhancement in various conditions of its operation and measurement.

The paper is structured as follows. Section 2 presents materials used in studies, in Section 3, the proposed method is explained. Section 4 discusses the experimental results, followed by the final conclusions in Section 5.

### 1.1. Characteristics of Thermal Cameras in Terms of Their Calibration

A thermal camera consists of a focal plane array and lens, both of which have a specific influence on the acquired images. The main parameters of the thermal camera are characterized below. 

#### 1.1.1. Structure of the Camera

The main element of the structure of a thermal camera is a detector that converts the infrared energy (IR) to other physical values, such as voltage, current, and the emergence of electric charge or change in resistance. Such a conversion of radiation energy into an electric signal enables us to determine the temperature of an object corresponding to a blackbody. However, only considering other properties of the object (such as emissivity, absorption, resistance) and the influence of the environment makes it possible to calculate the actual temperature. Commercially available products include those based on cooled detectors and those equipped with uncooled micro bolometric detectors. Another essential parameter that is connected with the detector is thermal sensitivity [50]. It is the lowest distinguishable difference in temperature that the camera is able to detect. The metrological parameter that determines the observation capacity of a thermal camera is the minimum resolvable temperature difference (MRTD) [51]. The test is conducted on a target consisting of four poles of varying distances between the gaps between individual bars. In practice, the test consists in heating the bars until the observer starts to distinguish all of them on a uniform background. MRTD links the spatial resolution to thermal resolution and measures the influence of noise on these parameters. A similar method used to determine the observational characteristics of cameras is the minimum detectable temperature difference (MDTD) [52]. The difference is the shape of the test: instead of bars, black objects in the form of circles or squares are used. Simple, commercially available cameras offer a thermal sensitivity in the range of 0.1–0.2 °C. If it is necessary to conduct measurements with much higher sensitivity, the market offers equipment with cooled detectors, which are commonly used to locate damages of photovoltaic panels.

#### 1.1.2. Field of View and Resolution of the Camera

Due to their specific construction, thermal cameras are equipped with fixed focal length lenses, which prevent using so-called optical zoom. When recording thermal images, one should strive for the object to fill the maximum possible part of the field of view.

Most commercially available cameras are equipped with rectangular focal plane arrays, which means that the horizontal and vertical angles of view are different. Apart from filling the maximum part of the image, one should also remember the relation between the distance and sharpness of the image. For low-angle lenses, this distance should not be less than 1.2 m.

#### 1.1.3. Spatial Resolution–IFOV

Spatial resolution, which is also sometimes referred to as the instantaneous field of view or IFOV, defines the dimensions of the smallest object that may be registered by a single-pixel (detector) [53]. This value depends on the resolution of the detector matrix and the field of view (1):

(1) $$\frac{\mathrm{lens}\;\mathrm{angle}\;\mathrm{of}\;\mathrm{view}\;\left[{}^{\circ}\right]}{\mathrm{camera}\;\mathrm{resolution}}\cdot \frac{2\pi }{360{}^{\circ}}\cdot 1000=\mathrm{spatial}\;\mathrm{resolution}\;\left[\mathrm{mrad}\right]$$

In technical specifications, the IFOV parameter is denoted in angular measurements (milliradians). The measurement of a specific detector is referred to as the theoretical ratio of the distance to the size of the measurement spot [54,55].

(2) $$\mathrm{FOV}\;\left[\mathrm{mrad}\right]\cdot \mathrm{distance}\;\left[\mathrm{mm}\right]=\mathrm{IFOV}\;\left[\mathrm{mm}\right]$$

Similar to pixels, detectors are always square. In order to obtain a correct temperature measurement, the section described above must be completely filled with the area of the object. The resulting temperature value is always the average value in the area of the section throughout its surface area. A single detector may enable presenting temperature differences on the thermogram, but, due to the phenomenon of optical dispersion of radiation, it is unable to provide a sufficient amount of energy that will allow for obtaining a reliable result. 

Determining the temperature distribution correctly is possible only for objects that are three times the size of a single converter. When used in calculations, the IFOV should be multiplied by three.

The calculations for the camera are presented below:

- Field of view: 32° × 26°;
- Camera resolution: 640 × 512 [px].

### 1.2. Radiation Emissivity Coefficient

Emissivity (*ε*) describes the ability of the given object (or material) to emit IR. An object with a high-emissivity value will be easily measurable. A value of (*ε*) close to zero means that it will be difficult for the camera to measure radiation energy. The emissivity value depends on: the type and structure of the material, the angle of observation (*α*), the temperature of the object (*T*), and the wavelength (*λ*) [56,57,58,59]. Emissivity is expressed as the ratio of the radiation emitted by the analyzed object M(*T*) and the radiation emitted in the same conditions by a blackbody *M*_0_(*T*):

(3) $$\varepsilon =\frac{M\left(T\right)}{M_{0}\left(T\right)}$$

## 2. Materials

The discussed experiment was carried out with a DJI Phantom 3 Standard, Unmanned Aerial Vehicle a FLIR VUE PRO 640 R thermal camera, and test fields developed by the authors. The concept involves the construction of a low-cost, passive, flat 2D test field that consisted of a high-emissivity background and low-emissivity targets using the phenomenon of thermal conductivity of certain materials. 

### 2.1. Characteristics of Thermal Cameras

**Figure 1:**
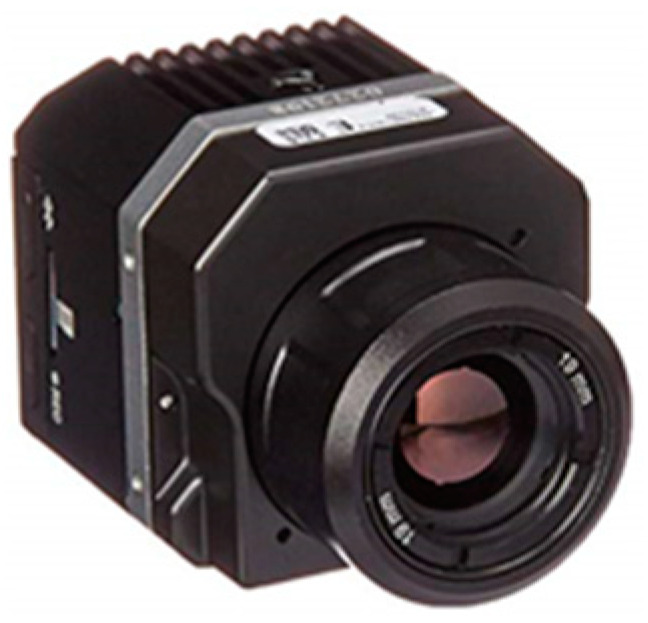
FLIR VUE PRO thermal camera.

The FLIR VUE PRO 640 R (Figure 1) is a high-resolution thermal camera that may be an integral part of Unmanned Aerial Systems. Being equipped with a radiometric system, it is able to record and measure temperature from low altitudes.

The camera operates in the second infrared window. Because of the uncooled, microbolometric converters, it is lightweight and small. The camera is compatible with the MAVLink protocol, which enables the recording of data that are necessary to develop a photogrammetric product. The configuration of the protocol allows it to collect data on the GPS, IMU, GPS status, and flight altitude, which enable aerial terrain mapping, at regular intervals. The additional signal modulator (Pulse Width Modulation–PWM) ensures continuous communication between the unmanned platform and the ground control station (operator), so the camera operation (the color palette, start/end of recording, or digital zoom) may be controlled remotely. The technical specifications of the camera are presented in Table 1.

### 2.2. Mounting of the Camera on UAV

Thanks to it being lightweight, the camera may be mounted on any UAV. The commercially available stiff suspensions and camera stabilization systems, such as SKY EYE-II GIMBAL are compatible with the most popular DJI drone series and others. For the purposes of the experiment, the mounting system was prepared by the authors from a 0.5 mm thick stainless steel sheet. The suspension is compatible with the DJI Phantom series. The spacing and diameter of holes enable the mounting of the handle to the mounting plate with the use of anti-vibration mounts (shock-absorbing rubber elements) to reduce the vibrations caused by the rotors. The camera itself was mounted with 3 M2 × 0.4 screws. Two of them support the camera, while the third one enables changing the tilt angle in the range of 0 ÷ 90°.

Thermal cameras are extremely sensitive to changes in electric current and the possibility of reverse polarization [30,61]. The device was created with the aim to be integrated with several series of vertical take-off and landing drones, so the suspension was equipped with an independent power source that provides energy for 120 min of flight. The whole set including battery and wiring weighs 122 g.

**Figure 2:**
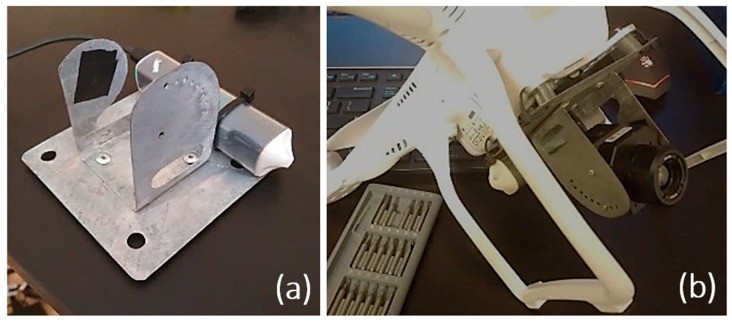
(**a**) Camera mounting unit (**b**) UAV with mounting unit and camera used in the experiment.

Mounting the thermal camera required removing the standard sensor together with the image stabilization system (Figure 2).

While obtaining data, the platform was at a fixed altitude of 15 m above ground level, which allowed us to obtain a Ground Sampling Distance (GSD) of 1.3 cm. Five side and cross stripes were planned in the flight area, to provide 90% overlap. In order to minimize the influence of the camera’s temperature on the measurement of the actual temperature of the object, it was switched on 15 min before the start of flight to ensure temperature stabilization. All thermograms were captured with the optical axis of the camera in vertical position. The duration of the flight was 9 min.

### 2.3. Calibration Target and Pattern

The quality of the calibration target is one of the main factors affecting the accuracy of calibration [62,63]. The main elements of the test field are the pattern, the size, and the material from which the field is made.

#### Selection of Materials to Construct Passive Calibration Fields

**Figure 3:**
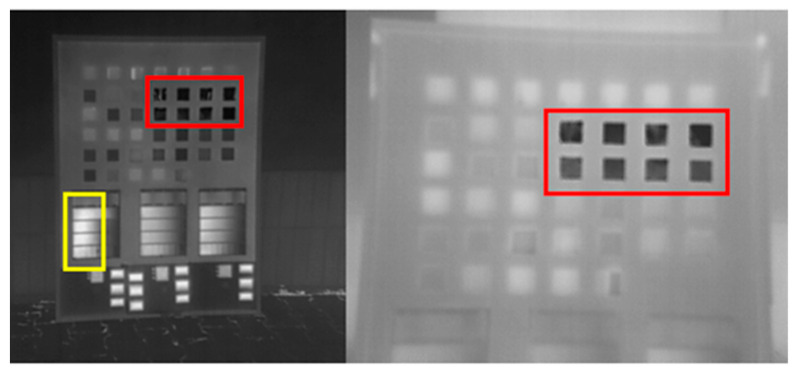
Thermogram of the board with material samples.

The first stage of the tests involved constructing and analyzing a special board, on which 40 types of materials were placed (e.g., matte and polished aluminum, cork, fabric, etc. (red rectangle)) as well as 6 samples of paper of various reflectance and physicochemical properties. All the materials are generally available and easy to process, but they differ in terms of emissivity. The board with test elements (Figure 3) was captured with the FLIR VUE PRO R thermal camera, obtaining the results presented below.

**Figure 4:**
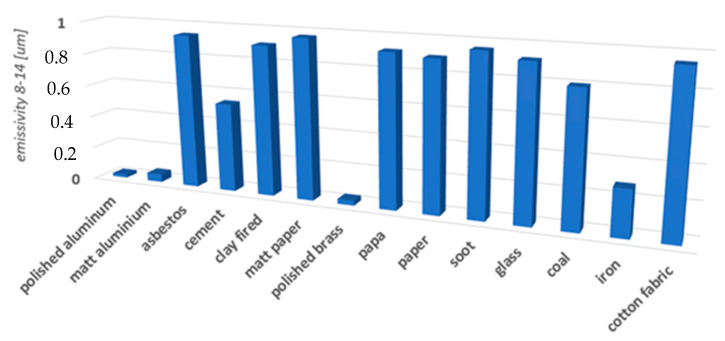
Table of emissivity of selected materials (8–14 μm).

The red rectangle marks metals, which were characterized by the lowest reflectance. Matte paper placed in the bottom part of the board reflects electromagnetic radiation very weakly (yellow rectangle). The differences in emissivity between the paper and printed ink are too small to ensure appropriate contrast of thermograms. The comparison of emissivity values with the obtained results in the image (Figure 4) allowed for the selection of suitable materials.

**Figure 5:**
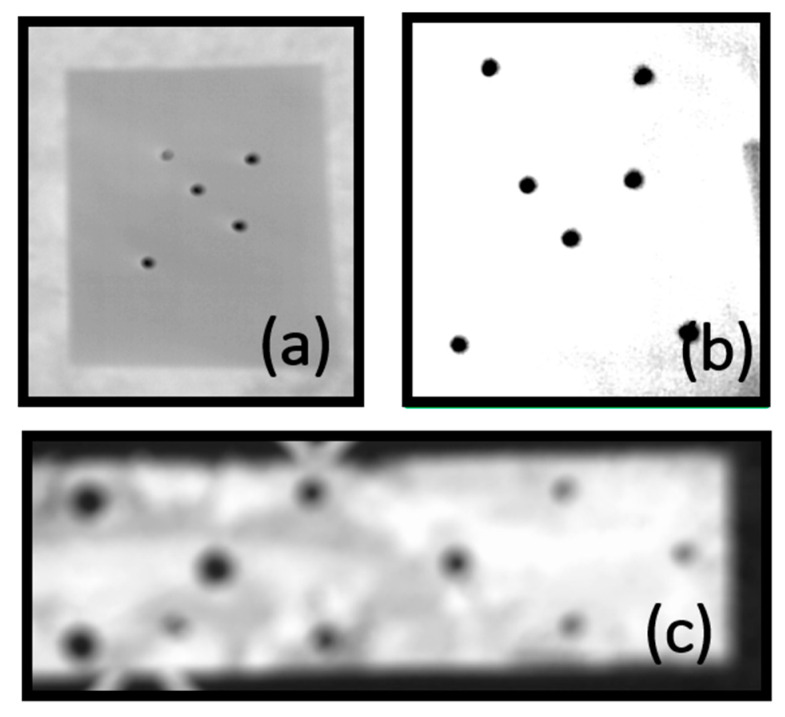
Passive 2D calibration tests made from various materials: (**a**) presents the visibility of low-emissivity aluminum targets on high-emissivity background (paper) in thermal image; (**b**) the visibility of low-emissivity targets (aluminum) on high-emissivity cotton fabric; (**c**) the visibility of high-emissivity targets on low-emissivity sheet aluminum in which holes of a diameter of 10, 12, and 16 mm were drilled (as a result of high reflectance coefficient of sheet metal the point targets were blurred).

Calibration targets were constructed from shiny aluminum foil and polished sheet aluminum, while the background of the chart was made from matte photosensitive paper and matte cotton fabric (Figure 5). Ultimately, cotton fabric of an emissivity coefficient = 0.97 was used. However, the selection of the material to prepare the targets proved to be more of a problem. Both sheet metal and foil reflect electromagnetic radiation well—they have a low emissivity coefficient.

Ultimately, calibration boards were constructed from a cotton background with an emissivity coefficient of 98% and aluminum targets with an emissivity coefficient of 4%. The aluminum foil, which plays the role of the calibration targets, leads to the emergence of dispersed radiation which is recorded by the camera. On the other hand, the emissivity coefficient of cotton fabric is high, so it emits only radiation resulting from its own temperature, such as a blackbody. Considering the above, the image of the calibration board should be highly contrasted, with very dark point targets and a bright background.

**Figure 6:**
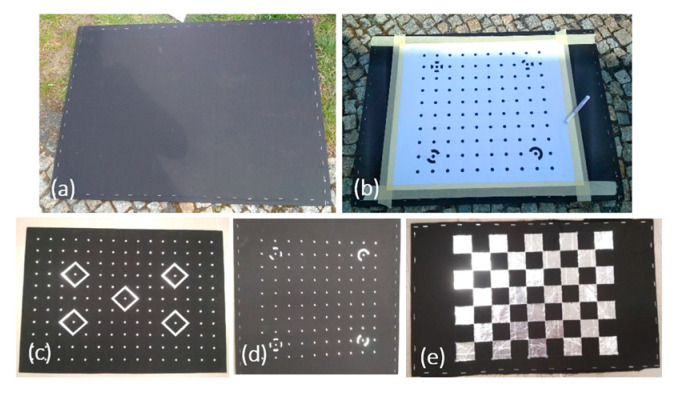
Subsequent stages of constructing calibration fields: (**a**) board–background; (**b**) board with a pattern of calibration targets; (**c**–**e**) boards dedicated for various software products.

Passive test fields were prepared for four types of calibration target patterns. The patterns are dedicated to different algorithms calculating the elements of the interior orientation of the camera, implemented in various software. The subsequent stages of constructing test fields are presented in Figure 6.

Phase I requires preparing stiff support for the calibration board. Phase II requires developing calibration targets for Image Master (Figure 6c), PhotoModeler Scanner (Figure 6d), Matlab (Figure 6e). Test fields for Matlab and PhotoModeler were enlarged, respectively, to A3 and A1 formats. The estimated error resulting from the potential displacement of calibration target is ±1 mm. This accuracy is acceptable for fast calibration tests. The distance from the camera to the calibration board is related to the software, camera IFOV and GSD (Table 2).

Based on the above table, the distance between the thermal camera and the calibration test was equal to 2 m; thus, GSD was equal to 1.7 mm. Since the diameter of the point targets on the passive 2D calibration test were equal to 10, 12, and 16 mm, the conditions that the dimensions of the acquired object should be at least three times larger than the GSD were met.

**Figure 7:**
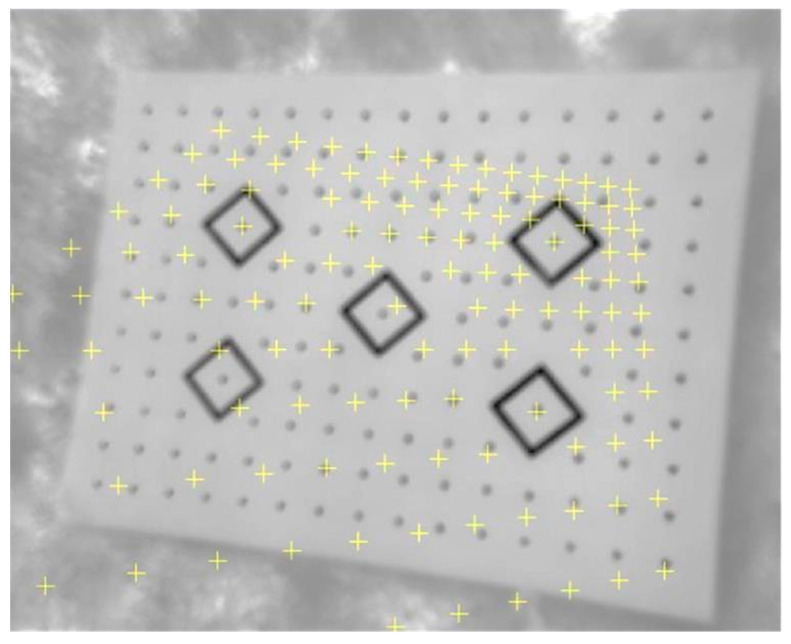
Raw, uncorrected test image with misidentified targets.

In many cases of automatic detection, the calibration target in the raw uncorrected image meets some difficulties and gross errors (Figure 7). 

Therefore, the authors proposed methods of image quality improvement, as described below. 

## 3. Methods

**Figure 8:**
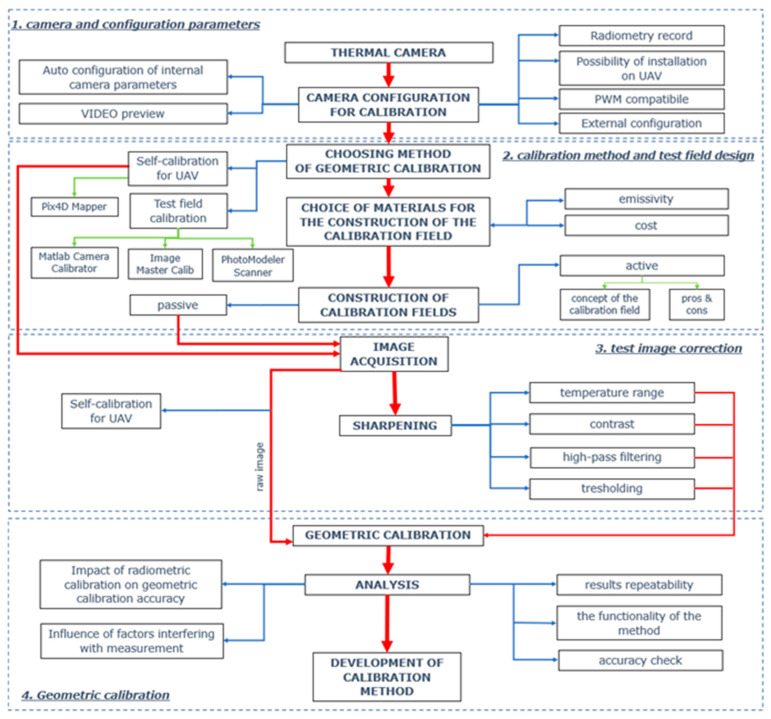
Overview of the proposed methodology.

The proposed methodology is based on a test field that is correctly designed, both in terms of emissivity and the shape and structure of its elements. The proposed methodology comprises four main steps, as shown in Figure 8.

The first step is camera configuration and preparation. Radiometric calibration, interior parameters, and camera installation are prepared. The second step is the design and selection of a test field that will be suitable for the selected method of geometric calibration. A properly structured and prepared test field will provide information and image data for geometric calibration. This stage is very important due to the fact that the aim of the method is to conduct a highly accurate calibration based on possibly low-cost test fields. In parallel, an active calibration test concept was developed that is beyond the scope of this article.

The third step is image acquisition and processing. The obtained raw imagery is processed to support accurate test feature extraction. The fourth step was to define the influence of various acquisition conditions of the calibration tests on their quality in the further geometric calibration of the camera. This will ensure appropriate outputs to be used for further image processing and generating products (3D models, orthophotomaps). Apart from calibration, this step also involves the analysis of the influence of factors interfering with the measurement, functionality of the method, or its accuracy.

The proposed methodology relies on capturing and appropriately processing the vertical image data by the process of fast calibrating the sensor dedicated for UAV applications in order to obtain the highest possible accuracy for low-cost cameras and other solutions (platforms). 

**Figure 9:**
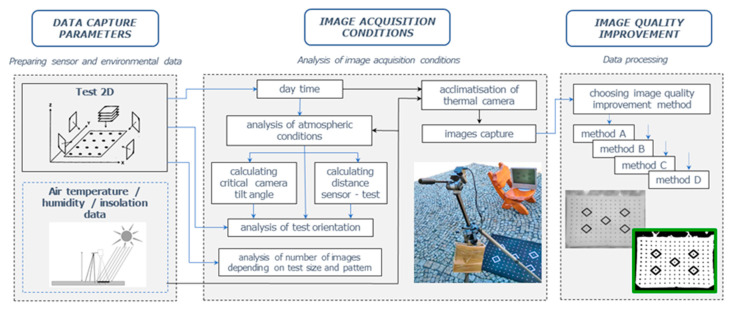
Methodology of selection of appropriate parameters for obtaining 2D test images for UAV thermal cameras.

The illustration below (Figure 9) shows a study of the selection of appropriate parameters for obtaining 2D test images for UAV thermal cameras.

The test was conducted in several variants: variable weather conditions, different times of day, and board orientation. Another aim of the test was to determine a critical angle, at which point targets would become insufficiently visible, so various angles of camera tilt were tested. All measurements were taken on a tripod, with a vertically oriented optical axis.

### Thermogram Quality Enhancement–Image Processing

One of the stages of the geometric calibration of the camera is the detection of features of the calibration tests. At this stage, image quality is essential as it will determine the possibility of automatically detecting image properties. The spatial resolution of thermal images is usually lower than that of visible spectrum cameras. Combined with the radiometry characteristic for thermal cameras, this results in low contrast and thus low image quality.

**Figure 10:**
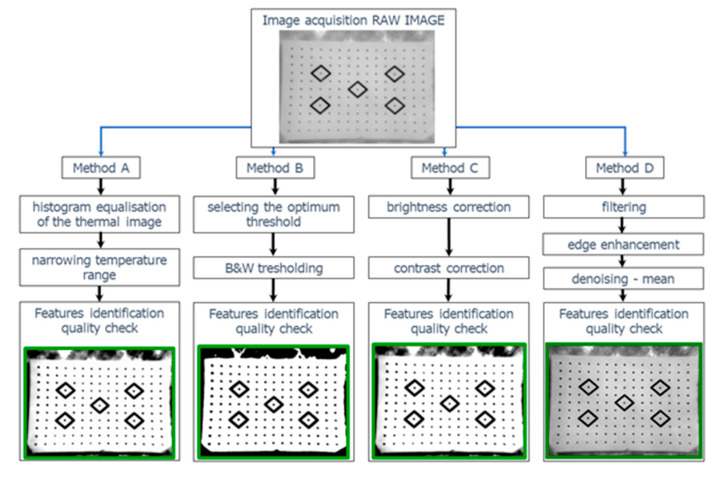
Methods of image processing flow.

A good way to enhance image quality is to use digital image processing methods, such as in [64,65], or using super-resolution algorithms [66]. A low range of changes in pixel brightness means that the sharpness/contrast of the image may be low. Using materials of high and low emissivity coefficients certainly allowed us to improve the thermal contrast consisting of general information about the experiment of improving thermogram quality that will allow the users to perform fast calibration in field conditions. Figure 10 presents a flowchart of the proposed methods of the image processing flow. 

In the course of the research, the authors developed four methods of enhancing feature contrast between the background and calibration targets.

**Figure 11:**
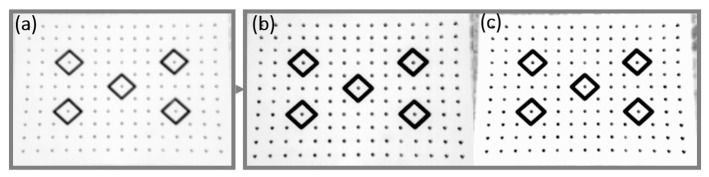
Contrast enhancement with use of histogram equalization method: (**a**) image without correction; (**b**) histogram equalization; (**c**) narrowing the temperature range.

Method A. Temperature range adjustment. The method is based on the knowledge of temperature distribution in the image. It was created with a view of thermal cameras that are capable of radiometric recording. Considering cheaper versions of cameras, such as VUE PRO, contrast enhancement methods should be used based on pixel density. The possibility to save information about the radiometry of each pixel in the image allows us to enhance contrast by means of expanding the temperature display range. Thermal images may be described with the use of a histogram that presents the share of pixels of specific temperatures (not brightness) in the image. Contrast enhancement based on subjective criteria may also be performed by modifying the histogram. Image correction based on simple, single-point operations, results in changing the shape of its contour. Initial contrast enhancement may also be obtained by the equalization (linearization) of the histograms. The illustration below (Figure 11) presents the effects of enhancing the contrast of a thermogram.

For control purposes, two other variants of contrast enhancement were also analyzed with the aim to check whether there is a safe range of enhancing the sharpness with the use of the method described above. In the first attempt, the scope of the range was defined as 1 °C, and in the second −0.1 °C. The analysis of the modified thermograms did not reveal any correlations between the range of displayed temperatures and image sharpness. Corrections should be made individually for each image, defining a range that will make it possible to distinguish the calibration targets from the background to the maximum possible extent.

**Figure 12:**
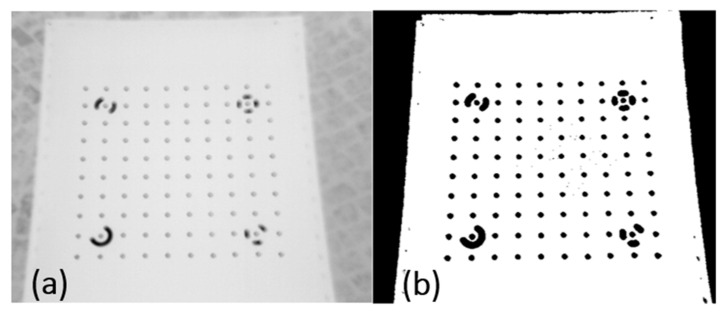
Contrast enhancement with the use of the thresholding method: (**a**) raw image; (**b**) image after thresholding.

**Figure 13:**
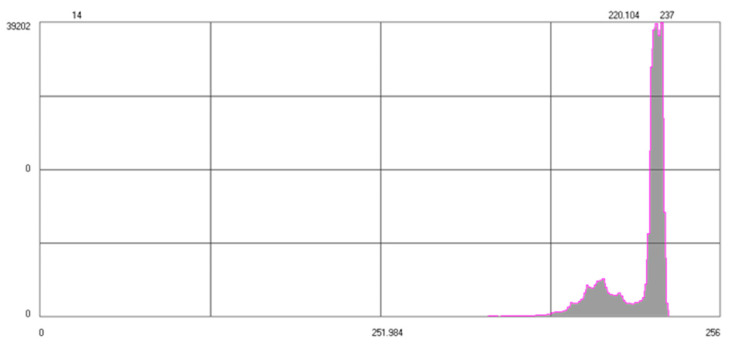
Histogram of the thermogram.

Method B. Image quality enhancement with the use of the global thresholding method. The selection of an optimum threshold that would enable segmentation, i.e., dividing the pixels in the image into two classes (targets and background, Figure 12) is a very difficult task. The analysis of the histogram revealed that it had a bimodal distribution, i.e., that it was possible to define a minimum between the peaks (Figure 13).

The disadvantage of segmentation is the lack of guarantee that pixels that belong to the same group will be located next to each other creating a consistent image. Although the results are not always completely satisfactory, the method is widely used due to its simplicity and speed.

It was noted that the algorithm correctly divided the elements of the calibration field into targets and background. However, it should be emphasized that in order to apply this technique, the objects in the histogram area have to be separate. The threshold is set manually based on the analysis of a specific series of images.

**Figure 14:**
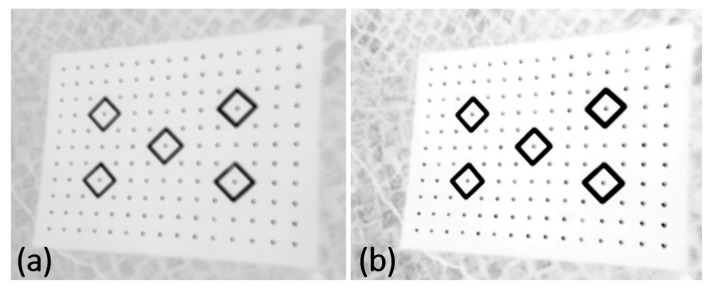
(**a**) raw image (**b**) sharpness enhancement by adjusting brightness and contrast.

Method C relies on quality enhancement by modifying brightness and contrast. In many cases, appropriate bi-parametric image processing sufficiently allows for the enhancement of image sharpness. In contrast to the thresholding method, the resulting image still consists of 256 shades of grey, while thresholding results in creating a binary image (Figure 14).

The method enhances the image without normalizing the information contained in it, so the image does not lose its informational value despite the transformation.

Method D. High-pass filtering. Digital filters belong to the category of spatial operations. The structure of the tool is more complex than that of single-point transformations because the new pixel density is determined with the use of a multi-argument function that takes into account the values of neighboring pixels–the range of pixels depends on the size of the mask. The authors used an adaptive filter characterized by the capacity for non-directional detection of point and line objects. Its structure resembles that of Laplacians, but due to its two-step operation, it does not cause an increase in background noise.

## 4. Results and Discussion

This section presents the experimental results for all datasets and test pattern types. Each “image acquisition stage”, regardless of the dataset examined, was obtained via three or four measurement sessions. 

We discuss the influence of external factors on the quality of the calibration test’s image. Conducting an accurate thermal measurement requires taking into account numerous factors that may interfere with the process and introducing the relevant adjustments to the obtained results. The following section presents an analysis of the influence of physical, methodological, and measurement-related factors on the results of the calibration image quality improvement for a UAV thermal camera.

### 4.1. Influence of the Working Temperature on the Contrast of Thermal Images–Acclimatisation

Quick heating and cooling of the detector matrix result in unreliable temperature values. Measuring the actual temperature of the object is not necessary for geometric calibration, as the algorithms for the detection of target points are based on pixel relative values. The aim of the experiment was to verify the existence of a relationship between the working temperature of the camera and the contrast visible on the thermograms. One measurement series was intentionally started immediately after switching on the device in order to prevent camera acclimatization. Ten seconds after starting the measurement, the camera was forced through the application to recalibrate the detector temperature. The recalibration was performed on image No. 4 (see Table 3). 

As a result of heating, the camera generates inaccurate temperature values. This test allowed us to evaluate the reliability of the system responsible for the stability of the recorded object temperature.

### 4.2. Influence of Image Processing on the Precision of Detecting Calibration Targets

Digital image processing methods (Figure 4) have a tendency to enhance contrast and to improve the ability to detect targets in the calibration process. Table 4 presents the results for images with correct automated detection of the calibration targets.

The (A, B, and C) processing methods resulted in over 80% effectiveness, respectively, on average: 94%, 81%, and 80%. This effectiveness for no processing and processing with the use of the D method was, respectively, 42% and 38%. Only high-pass filtering did not improve the obtained results. Despite its two-stage operation, the applied mask introduced local noise that disturbed the operation of the algorithm responsible for detecting targets. Unfortunately, any interference with image structure carries a risk of changing the measured physical parameters that are closely linked to the determined elements. Apart from testing the effectiveness of identification, the authors also analyzed the influence of enhancing contrast with the use of image processing methods on the stability of calibration. The tests were conducted using the image enhancement method based on changing brightness and contrast (method C). Three sets of images were subjected to two enhancing operations with defined different values of the parameters of brightness and contrast. Table 5 presents the overall results for the selected parameters of interior orientation.

The analysis of the average values reveals that contrast enhancement influences the determined parameters of interior orientation. The biggest differences were noted for distortion, which, apart from the order of magnitude, also changed their value from positive to negative and vice versa. While analyzing the matrix, the algorithm seeks point extremes that most likely determine the position of calibration targets. The operation of this algorithm resembles that of high-pass filtering operating based on a pre-defined mask. Starting the sharpness adjustment process, one should bear in mind that too strong interference with the structure of the image may lead to an unintentional displacement of points during calibration. This may be the main reason for such large divergences for radial and tangential distortion errors. The above relationship applies to all the used methods of digital image processing.

### 4.3. Influence of Manual and Automated Enhancement on Calibration Accuracy

An experimental test of the influence of automation on the quality of contrast enhancement was also conducted based on the results of image correction with the use of the histogram equalization method (method A). The automation involved the need to apply fixed correction parameters to all images, which resulted in the deterioration of the interpretation capacity of some of them. Three sets of input data were subjected to processing. First, the data were processed automatically. Then, each image was adjusted manually. The accuracy analysis was based on the values of the determining elements of interior orientation. Detailed results are provided in Table A1–Appendix A, while Table 6 below presents the standard deviation for both methods and the elements of interior orientation.

The analysis of standard deviation values reveals that the results of the manual adjustment of image quality enhancement parameters for the determined elements are nearly two times better than for automated correction. Future research on the geometric calibration of thermal cameras should involve developing an algorithm that would select the sharpness enhancement parameters for each image individually, based on histogram analysis.

### 4.4. Influence of Atmospheric Conditions on the Contrast of Thermograms

The aim of the study was to determine the optimum measurement conditions that would enable obtaining the best quality of thermal images. Thermograms were captured in the following conditions: in a shady area on a sunny day, sunny area on a sunny day, on a cloudy day, and at night. Image contrast was evaluated by measuring the difference in temperatures between the calibration targets and the background. Eleven thermal images were selected for analysis–all of them met the condition that the target axis of the camera should be oriented normally to the calibration field. In each image, nine temperature measurements were taken: five on calibration targets and four on the sheet background. Detailed results are presented in Table A2–Appendix B. Table 7 presents the average values for all results.

**Figure 15:**
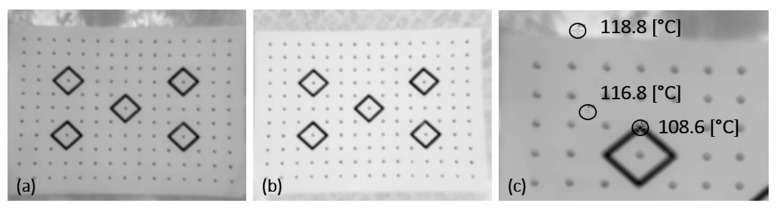
Visual analysis of the sharpness of thermograms: (**a**) daytime, shady area; (**b**) daytime, sunny area; (**c**) value measurement on the thermogram.

Measurements taken in direct sunlight result in the sharpest images. According to the Kirchoff law, high-emissivity fabric absorbs almost all the energy of solar radiation, which causes an increase in its temperature. On the other hand, aluminum, with its high reflectance coefficient, which corresponds to a perfect white body in the test, reflects nearly 100% of the energy supplied to its surface. The conducted visual analysis suggests that the sharpness of images captured during daytime in shady areas is better (Figure 15a) than of those taken in sunny areas (Figure 15b).

This false impression is caused by the lack of an analysis of the influence of soil temperature on the contrast between the targets and the background. Soil, like fabric, has a high emissivity coefficient of approx. 0.96. As the soil was exposed to direct solar irradiation for a longer time, its temperature was higher than that of the cotton background of the board. The result was a decrease in contrast on the calibration field (Figure 15c). 

For outdoor calibration, it is recommended to select a base that does not accumulate a large part of the heat energy radiated on its surface. To prevent this phenomenon, a material that will not interfere with the thermal contrast of the calibration field may be placed below the test field. The emissivity coefficient of such material should be higher than that of calibration targets and lower than the emissivity of cotton fabric.

### 4.5. Influence of Calibration Test Field Orientation on Image Quality and Calibration Accuracy

**Figure 16:**
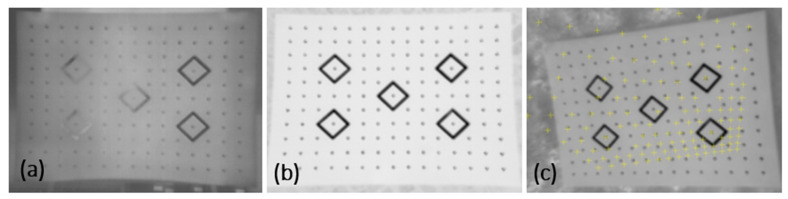
Thermal contrast of thermograms for: (**a**) vertical; (**b**) horizontal orientation of the calibration board; (**c**) incorrect target identification at a too large camera tilt angle.

The aim of this test was to analyze the influence of the calibration board on the accuracy of the reflected radiation measurements. This is connected, among others, with the critical angle of camera orientation towards the calibration board and the sensor-test distance. For the purposes of the experiment, a series of images were captured in the same measurement conditions, but at different orientations. According to the analysis presented in Section 1.1, in order to obtain the optimum conditions for total energy measurement, the capturing distance should be selected so as to ensure the ratio of resolution 3 × IFOV. Image data were obtained from the 2 m altitude (the whole board is visible). Figure 16a,b present the differences in thermogram contrast for vertical and horizontal positions of the calibration board.

The analysis of the images above reveals that the interference in the thermogram of a vertically oriented board is stronger. This results from the fact that radiation originating from neighboring objects was captured, which is one of the factors interfering with thermal visual measurements. Due to that, it is recommended to place the calibration fields flat on the ground to ensure direct solar irradiation, away from objects that may additionally reflect radiation. The best time to capture thermal images is the moment when the sun is at its highest point. The best results for reflected radiation are obtained at the direction normal to the plane of the calibration field. The camera tilt angle must not exceed 10°. Board orientation directly influences the geometry of the images selected for calibration and it may influence the correct detection of calibration targets (Figure 16c) and thus also calibration accuracy. The influence of the geometry of images with correctly and incorrectly identified calibration targets was tested based on four measurement series. Due to the fact that contrast enhancement methods improve the accuracy of the geometric calibration, images of the Image Master calibration board were used in the experiment. Their contrast was enhanced by histogram equalization and by adjusting the range of displayed temperatures. For each measurement series, calculations were performed twice. In the first iteration (I), all images were imported, including geometrically incorrect ones. The second calculation (II) was performed only on those images that met the set geometry conditions. Table A3, containing the detailed results of the analysis of the influence of image geometry on calibration accuracy, is provided in Appendix C. Table 8 presents the overall list of standard deviations for both measurement series.

Such large divergence values of errors are due to the lack of some targets, or due to the incorrect determination of their position by the software. 

Calibration accuracy decreases nearly by half if images with incorrect geometry and contrast are taken into account. Before starting calibration, it is recommended to select and remove the images that do not meet the criteria concerning their orientation. 

Calibration was carried out with the use of various software, based on different calibration target detection algorithms and different test field patterns.

The methodology of capturing images for the Image Master software required modifications due to the discrepancies related to the orientation of the calibration field: according to the instruction manual of the software, the board should be attached to a wall. The results presented below allowed us to analyze whether the new concept, combined with digital image processing methods, allows us to improve the calibration accuracy. Detailed results of geometric calibration for three measurement series and different image quality enhancement algorithms are presented in Table A4, Table A5, Table A6 and Table A7 in Appendix D. An overall list of the accuracy of the determined parameters is provided in Table 9.

The results presented in Table 9 show the differences in the accuracies (SD) of the interior orientation parameters depending on the method. The best improvement was obtained from the methods of histogram equalization and brightness adjustment (two to three times better results).

The same set of thermograms was used in all the analyses for each conducted calibration. Diagonal images were excluded from the tests as it was impossible to identify calibration targets correctly, especially in images not subjected to sharpness enhancement. The value of the standard deviation for the fixed focal length of principal point displacement fluctuated around 0.1 mm. The order of magnitude of the exponents for distortion was similar in most cases. What is disturbing is the fact that the same distortions could take opposite values, although the order of magnitude of the exponent remained similar.

The calibration board dedicated for Camera Calibrator for Matlab consists of alternating high- and low-emissivity elements, which allows us to avoid the phenomenon of optical dispersion of radiation. Due to the simplicity of construction, the calibration field enabled the correct detection of calibration targets on almost all thermograms, regardless of the observation angle. Table 10 presents the effectiveness of the algorithm responsible for detecting the corners of squares.

Calibration images were captured in two variants–for a moving camera and a moving test. The obtained results demonstrated that the movement of the camera during calibration makes it impossible to determine the elements of interior orientation correctly as the results did not repeat. However, in the approach where only the test was moving, the stability of the obtained results was maintained. Test fields constructed in this way ensure highly stable and accurate results of geometric calibration of thermal sensors that are characterized by low resolution and sharpness of thermograms. 

## 5. Conclusions

This paper presents a methodology for a low-cost and fast geometric calibration of the thermal UAV FLIR VUE PRO camera. The aim of the study was to develop a universal method of improving thermogram quality that will allow users to perform calibration in field conditions for this camera. 

The main aim of applied research was to find an algorithm for processing the thermal image of the calibration test that would enable the correct geometric calibration of the FLIR VUE PRO camera. Therefore, all analyses in Section 4 relate to the analysis of the influence of all parameters including working temperature, atmospheric conditions, manual and automatic image enhancement, etc., on thermal image quality. The thermal images on which the calibration tests are located are in turn used to perform the geometric calibration. Thus, the significance of the content of the applied research and results intersects with the issue of geometric calibration quality. It is difficult to unambiguously determine the level of the influence of the individual parameters, but the algorithms proposed by the authors for processing thermal images make it possible to counterbalance them.

The tests revealed that, apart from atmospheric conditions, the methods of enhancing the quality of the captured thermograms also play a major role. 

Algorithms based on a 2D checkerboard test field were nearly 100% effective in correctly detecting the calibration targets on thermograms that were not subjected to any sharpness enhancement. It was noted that the likelihood of detecting calibration points in images with an enhanced contrast was higher than in images that were not subjected to any graphic corrections. The best results were obtained from the following methods: histogram equalization and brightness and contrast adjustment. The selection of various image processing methods (histogram equalization, thresholding, brightness correction,) in order to improve the quality of the thermograms have been proposed. The consecutive processing methods resulted in over 80% effectiveness, on average: 94%, 81%, and 80%, respectively. This effectiveness for no processing and processing with the use of the filtering method was 42% and 38%, respectively. Only high-pass filtering did not improve the obtained results.

The differences in standard deviations of the interior orientation parameters are two to three times better than for images without corrections. This applies in particular to thermograms with a large tilt of the optic system from the direction normal to the calibration field. Such thermograms are characterized by the highest sharpness fade-out and contain the most information necessary for the correct determination of the elements of interior orientation. 

The comparison of the determining elements of interior orientation with the metrics of calibration provided by the manufacturer revealed certain discrepancies between the radial and tangential distortion results. These errors result from the irregularities in the low-cost calibration field. The cotton fabric stretched on a wooden board may be displaced and cause differences in the final results. While selecting the materials to prepare a calibration board based on the phenomenon of thermal conductivity, special attention should be paid to their chemical composition and structure. It is recommended to increase the diameter of calibration points in order to enable the thermal camera to record a sufficient amount of reflected radiation energy. Complying with the 3 × IFOV principle will allow for improving the interpretation capacity of the thermograms.

Future research will focus on improving calibration targets, constructing a three-dimensional calibration field test, and examining the impact of the target quality on geometric calibration for different calibration algorithms.

## Figures and Tables

**Table 1:** Technical specifications of the FLIR VUE PRO R 640 thermal camera [60].

**General Information**	
Sensor type	Uncooled Vox Microbolometer
Matrix resolution	640 × 512 [px]
Size of the smallest photosensitive element	17 [mm]
Focal length	19 [mm]
Field of view of the camera FOV	32° × 27°
Spectral range	7.5–13.5 [μm]
Refreshing (UE)	7.5 Hz (NTSC); 8.3 Hz (PAL)
Thermal sensitivity	<50 [mK]
Temperature measurement range	−55; +90 [°C]
Temperature measurement accuracy	±2 [°C]
**Physical properties**	
Dimensions with lens	45 × 45 × 63 [mm] (width) × (height) × (length)
Weight	110 [g]
Mounting	×4 M2 × 0.4; ×1 ¼−20 tripod thread
**Power supply and image display**	
Power source	External

**Table 2:** Relationship between IFOV and GSD calculated for FLIR VUE PRO thermal camera.

Distance [m]	0.5	1	2	5	10	15	25	50	100
GSD [cm]	0.04	0.09	0.17	0.44	0.87	1.31	2.18	4.35	8.70

**Table 3:** Influence of the working temperature of the camera on the accuracy of thermal measurement.

Image	1	2	3	4	5	6
Temp. target [°C]	109.2	112.5	113.2	108.4	109	107.8
Temp. background [°C]	116.5	115.8	115.7	115.9	116.6	116.6
∆	7.3	3.3	2.5	7.5	7.6	7.6

**Table 4:** Number of images with correctly automatically identified calibration targets.

Number of Images	Sharpness Enhancement Method				
	Raw Image	Histogram Equalization (Met A)	Thresholding (Met B)	Contrast and Brightness (Met C)	Adaptive Filter (Met D)
	Percentage of Correctly Oriented Images [%]				
20	45	85	75	60	40
23	30	100	95	96	30
25	52	96	72	80	44

**Table 5:** Differences in the determined parameters of interior orientation.

Measurement Series	Fixed Focal Length [mm]	PPA [mm]		Radial Distortion		Tangential Distortion	
		X	Y	R1	R2	T1	T2
I	18.860	5.245	4.003	−1.24 × 10^−3^	6.45 × 10^−7^	−6.06 × 10^−5^	−3.93 × 10^−4^
II	19.028	5.210	4.140	−1.21 × 10^−3^	1.11 × 10^−6^	1.61 × 10^−4^	−2.17 × 10^−4^
∆	−0.168	0.035	−0.138	2.20 × 10^−5^	−4.69 × 10^−7^	−2.21 × 10^−4^	−1.76 × 10^−4^

**Table 6:** Presentation of results for the automatic and manual methods (standard deviations).

Sharpness Correction	Fixed Focal Length [mm]	PPA [mm]		Radial Distortion		Tangential Distortion	
		X	Y	R1	R2	T1	T2
Automatic	0.092	0.206	0.277	4.36 × 10^−5^	8.22 × 10^−7^	3.27 × 10^−4^	5.77 × 10^−4^
Manual	0.055	0.010	0.131	3.55 × 10^−5^	5.13 × 10^−7^	3.23 × 10^−5^	1.70 × 10^−4^

**Table 7:** Average values for Table A7.

Measurement Object	Atmospheric Conditions during Measurement										
	Sunny, Measurement in a Shady Area [°C]			Sunny, Measurement in a Sunny Area [°C]				Night [°C]		Cloudy Day [°C]	
Calibration targets	91.1	90.5	107.4	108.9	109.7	109.1	109.1	92.3	110.8	90.2	107.0
Background	96.8	95.9	112.6	116.9	118.2	116.4	118.0	97.3	115.3	95.9	112.6
∆	5.7	5.4	5.2	8.0	8.5	7.4	8.9	5.0	4.5	5.7	5.6
Thermal contrast	5.5 [°C]			8.2 [°C]				4.8 [°C]		5.6 [°C]	

**Table 8:** Influence of image geometry on calibration accuracy (standard deviations).

Measurement Series	Fixed Focal Length [mm]	PPA [mm]		Radial Distortion		Tangential Distortion	
		X	Y	R1	R2	T1	T2
I	0.085	0.103	0.146	3.16 × 10^−5^	5.30 × 10^−7^	5.15 × 10^−5^	2.29 × 10^−4^
II	0.025	0.041	0.021	2.99 × 10^−5^	5.06 × 10^−7^	1.00 × 10^−4^	1.63 × 10^−4^

**Table 9:** Standard deviations for the results of specific methods of thermogram sharpness enhancement.

Correction Method	Fixed Focal Length [mm]	PPA [mm]		Radial Distortion		Tangential Distortion	
		X	Y	R1	R2	T1	T2
Without correction	0.22	0.13	0.13	8.32 × 10^−5^	7.72 × 10^−7^	3.06 × 10^−4^	2.44 × 10^−4^
Histogram equalisation	0.06	0.01	0.09	3.55 × 10^−5^	7.48 × 10^−7^	3.23 × 10^−5^	1.70 × 10^−4^
Thresholding	0.10	0.15	0.08	7.66 × 10^−5^	8.15 × 10^−7^	3.87 × 10^−4^	1.70 × 10^−4^
Brightness and contrast adjustment	0.09	0.10	0.04	6.04 × 10^−5^	5.30 × 10^−7^	2.74 × 10^−4^	1.05 × 10^−4^

**Table 10:** The effectiveness of the algorithm responsible for detecting calibration targets.

Series No.	1	2	3	4	5	6
Number of images taken	10	9	16	21	19	12
Number of accepted images	9	8	16	21	18	11
Result	90.0%	88.9%	100.0%	100.0%	94.7%	91.7%

## Data Availability

Data can be lent on request after e-mail contact.

## Appendix A

**Table A1:** Influence of manual and automated enhancing on geometric calibration accuracy.

Sharpness Correction	Fixed Focal Length [mm]	PPA [Mm]		Radial Distortion		Tangential Distortion	
		X	Y	R1	R2	X	Y
Automatic	19.00	5.29	3.69	−1.19 × 10^−3^	−2.95 × 10^−7^	−1.57 × 10^−4^	−1.14 × 10^−3^
Manual	18.99	5.36	3.99	−1.15 × 10^−3^	−3.22 × 10^−7^	−2.01 × 10^−4^	−4.65 × 10^−4^
Automatic	18.88	4.91	4.02	−1.24 × 10^−3^	1.38 × 10^−6^	6.47 × 10^−4^	−3.23 × 10^−4^
Manual	19.04	5.35	4.02	−1.17 × 10^−3^	2.20 × 10^−7^	−1.36 × 10^−4^	−4.03 × 10^−4^
Automatic	18.82	5.24	4.24	−1.28 × 10^−3^	1.91 × 10^−6^	2.65 × 10^−5^	−1.97 × 10^−5^
Manual	19.10	5.37	4.23	−1.22 × 10^−3^	1.16 × 10^−6^	−1.64 × 10^−4^	−1.44 × 10^−4^

## Appendix B

**Table A2:** Influence of manual and automated enhancing on geometric calibration accuracy.

Measurement Object	Atmospheric Conditions during Measurement										
	Sunny, Measurement in a Shady Area			Sunny, Measurement in a Sunny Area				Night [°C]		Cloudy Day [°C]	
Calibration targets	91.5	90.5	107.4	108.6	109.4	108.1	109.2	92.8	110.8	89.9	106.4
	90.3	91.0	107.3	108.1	109.7	109.4	108.9	93.2	111.1	90.4	107.3
	90.8	89.9	107.1	108.5	108.6	108.4	109.1	91.4	109.8	89.9	106.5
	91.8	91.0	107	108.5	110.6	110.2	108.7	91.6	112.1	90.9	106.5
	91.2	90.1	108.1	110.7	110.0	109.2	109.6	92.3	110.0	90.1	108.1
Background	96.8	96.0	112.6	116.8	117.9	116.2	117.9	97.2	115.2	96	112.4
	96.8	95.9	112.7	116.7	118.2	116.5	117.9	97.1	115.1	95.9	112.7
	96.8	95.9	112.5	116.9	118.4	116.5	118.4	97.3	115.3	95.9	112.5
	96.9	95.9	112.6	117.1	118.3	116.5	117.6	97.5	115.5	95.9	112.6

## Appendix C

**Table A3:** Analysis of the influence of image geometry on calibration accuracy.

Measurement Series	Fixed Focal Length [mm]	PPA [mm]		Radial Distortion		Tangential Distortion	
		X	Y	R1	R2	T1	T2
I	19.21	5.39	4.09	−1.20 × 10^−3^	−1.86 × 10^−8^	−1.29 × 10^−4^	−3.89 × 10^−4^
II	19.04	5.44	3.99	−1.17 × 10^−3^	−1.13 × 10^−6^	−3.60 × 10^−4^	−5.07 × 10^−4^
I	19.11	5.39	4.01	−1.13 × 10^−3^	−4.98 × 10^−7^	−1.90 × 10^−4^	−4.74 × 10^−4^
II	18.99	5.36	3.99	−1.15 × 10^−3^	−3.22 × 10^−7^	−2.01 × 10^−4^	−4.65 × 10^−4^
I	19.01	5.18	4.12	−1.16 × 10^−3^	−9.97 × 10^−8^	−2.21 × 10^−4^	−2.03 × 10^−4^
II	19.04	5.35	4.02	−1.17 × 10^−3^	−2.20 × 10^−7^	−1.36 × 10^−4^	−4.03 × 10^−4^
I	19.16	5.38	4.35	−1.19 × 10^−3^	−1.18 × 10^−6^	−1.11 × 10^−4^	7.54 × 10^−4^
II	19.04	5.37	4.03	−1.22 × 10^−3^	−1.16 × 10^−6^	−1.64 × 10^−4^	−1.44 × 10^−4^

## Appendix D

**Table A4:** Results of geometric calibration for three measurement series for raw images.

Time	Fixed Focal Length [mm]	PPA [mm]		Radial Distortion		Tangential Distortion	
		X	Y	R1	R2	X	Y
11:22	18.37	5.03	4.06	−1.38 × 10^−3^	1.09 × 10^−6^	2.22 × 10^−4^	−2.38 × 10^−4^
11:35	18.70	5.2	3.93	−1.33 × 10^−3^	2.07 × 10^−6^	−9.11 × 10^−6^	−5.79 × 10^−4^
12:16	18.79	4.95	4.09	−1.22 × 10^−3^	5.46 × 10^−7^	5.98 × 10^−4^	−1.05 × 10^−4^

**Table A5:** Results of geometric calibration for three measurement series for histogram equalization.

Time	Fixed Focal Length [mm]	PPA [mm]		Radial Distortion		Tangential Distortion	
		X	Y	R1	R2	X	Y
11:22	18.99	5.36	3.99	−1.15 × 10^−3^	−3.22 × 10^−7^	−2.01 × 10^−4^	−4.65 × 10^−4^
11:35	19.04	5.35	4.02	−1.17 × 10^−3^	2.20 × 10^−7^	−1.36 × 10^−4^	−4.03 × 10^−4^
12:16	19.10	5.37	4.23	−1.22 × 10^−3^	1.16 × 10^−6^	−1.64 × 10^−4^	−1.44 × 10^−4^

**Table A6:** Results of geometric calibration for three measurement series for thresholding.

Time	Fixed Focal Length [mm]	PPA [mm]		Radial Distortion		Tangential Distortion	
		X	Y	R1	R2	X	Y
11:22	18.99	5.27	4.05	−1.27 × 10^−3^	1.86 × 10^−6^	−3.85 × 10^−5^	−4.27 × 10^−4^
11:35	18.80	5.15	3.99	−1.20 × 10^−3^	8.54 × 10^−7^	1.29 × 10^−4^	−4.27 × 10^−4^
12:16	18.83	4.97	4.14	−1.12 × 10^−3^	2.47 × 10^−7^	7.00 × 10^−4^	−1.32 × 10^−4^

**Table A7:** Results of geometric calibration for three measurement series for brightness and contrast adjustment.

Time	Fixed Focal Length [mm]	PPA [mm]		Radial Distortion		Tangential Distortion	
		X	Y	R1	R2	X	Y
11:22	18.99	5.23	4.1	−1.27 × 10^−3^	1.48 × 10^−6^	5.66 × 10^−5^	−3.04 × 10^−4^
11:35	18.96	5.27	4.16	−1.17 × 10^−3^	6.47 × 10^−7^	2.83 × 10^−5^	−1.34 × 10^−4^
12:16	19.13	5.08	4.08	−1.17 × 10^−3^	5.00 × 10^−7^	5.16 × 10^−4^	−3.26 × 10^−4^
